# Refractory catatonia in old age: a case report

**DOI:** 10.1186/s13256-021-03000-3

**Published:** 2021-08-14

**Authors:** Emma Bean, Callum Findlay, Claire Gee, Jay Amin

**Affiliations:** 1 University Hospitals Southampton NHS Foundation Trust, Southampton, UK; 2 Memory Assessment and Research Centre, Southern Health NHS Foundation Trust, Southampton, UK; 3 Clinical Neurosciences, Faculty of Medicine, University of Southampton, Southampton, UK

**Keywords:** Catatonia, Electroconvulsive therapy, Benzodiazepines, Old age, Autistic spectrum disorder, Case report

## Abstract

**Background:**

Catatonia is a clinical syndrome characterized by psychomotor disruption, which often goes undiagnosed. Most reports have focused on interventions and outcomes for catatonia in younger people and those with schizophrenia. The clinical characteristics and course of catatonia in old age are poorly understood. We present a report of an older person whose catatonia was refractory to extensive treatment, and we identify important implications for the management of catatonia in old age.

**Case presentation:**

We describe a 73-year-old white man with longstanding autistic spectrum disorder who presented with symptoms of depression. Following a period of diagnostic uncertainty and failure to improve with antidepressant medication, a lorazepam challenge yielded an abrupt improvement in presentation. The patient was treated extensively with lorazepam, zolpidem, and electroconvulsive therapy during his 16-month hospital admission, but his catatonia ultimately proved refractory to treatment.

**Conclusions:**

Catatonia should be considered promptly as a differential diagnosis in older people presenting with atypical features of functional mental illness. Although partial improvement of catatonic features was achieved using benzodiazepines and electroconvulsive therapy, these were not sustained in our patient. We identified comorbid autistic spectrum disorder, prolonged duration of catatonia, and sensitivity to benzodiazepines as important factors in prognostication in old age.

## Background

The first descriptions of catatonia can be traced back to 1849 when Bell reported on 40 patients presenting with concurrent mania, delirium, psychosis, sleeplessness, and overactivity [1]. In 1874, Kahlbaum described catatonia as an independent psychiatric syndrome characterized by a cyclical course of alternating manic, depressive, and psychotic phases, with eventual deteriorating course [2]. While catatonia has historically been most closely associated with schizophrenia, it has more recently been recognized as a syndrome related to a range of psychiatric and medical disorders [3].

Catatonia is now defined as a clinical syndrome characterized by the coexistence of psychiatric and motor signs [4]. Criteria for catatonia are presented in the Diagnostic and Statistical Manual of Mental Disorders, Fifth Edition (DSM-5), and summarized in Table 1. Timely recognition of catatonia can be challenging, possibly due to poor awareness among some clinicians, or the longer period of observation often required for some catatonic signs to emerge [5]. Untreated catatonia can lead to a range of recognized complications, including infection, venous thromboembolism, aspiration pneumonia, and even death [6]. Catatonia is, however, very much a treatable condition with a response rate thought to range from 59% in patients with schizophrenia to over 90% in patients with other psychiatric diagnoses [4].

**Table 1:** Clinical diagnosis of catatonia

Clinical feature	Description
Catalepsy	Passive induction of a posture held against gravity
Waxy flexibility	Slight and even resistance to positioning by examiner
Stupor	No psychomotor activity; no reactivity to environment
Agitation	Agitation, not influenced by external stimuli
Mutism	No, or minimal verbal response
Negativism	Opposing or not responding to instructions or external stimuli
Posturing	Spontaneous and active maintenance of a posture against gravity
Mannerisms	Odd caricature of normal actions
Stereotypies	Repetitive, abnormally frequent, non-goal-directed movements
Grimacing	Maintenance of odd facial expressions
Echolalia	Repeating the words spoken by the examiner
Echopraxia	Mimicking the movements made by the examiner

DSM-5 criteria for catatonia [7], defined as the presence of three or more of the clinical features listed above

Benzodiazepines are the mainstay of treatment of catatonia [4, 6], with lorazepam the most used drug at a dose between 2 and 16 mg/day [8]. Indeed, catatonia can be confirmed with a lorazepam challenge test, where the patient is examined before and after a dose of 1–2 mg of lorazepam. A positive response is a swift and marked reduction in catatonic signs [6], which can be quantified using the Bush–Francis Catatonia Rating Scale [9]. While the precise mechanisms underlying the pathophysiology of catatonia are still poorly understood, it is hypothesized that it may be characterized by gamma-aminobutyric acid (GABA) receptor cortical dysregulation and deficits in the dorsolateral prefrontal cortex [10]. It is therefore postulated that benzodiazepines may act by increasing cerebral GABA signaling. Where treatment with lorazepam is not successful, or when adverse effects prevent use of therapeutic doses, electroconvulsive therapy (ECT) can also be highly effective [8].

Early recognition and treatment of catatonia generally leads to rapid resolution of symptoms [6]. However, the presentation, management, and prognosis of catatonia in old age is poorly understood, with only a handful of case studies published in this field to date [11–13]. In this report, we describe the case of an elderly catatonic patient who was ultimately refractory to treatment, raising important implications for the management of catatonia in old age.

## Case presentation

This 73-year-old white man had not previously been known to specialist mental health services and had in fact not seen his general practitioner (GP) for over three decades. His family reported that he had periods of depression during his late teenage years, when he left the merchant navy after only a few weeks, and in the third and fourth decade of his life. He neither sought nor received any treatment for these episodes. He never married, had no children, and did not work after leaving the navy. He lived alone following the death of his cohabiting parents and relied heavily on his siblings and nieces for assistance with food shopping and house maintenance. Notably, his mother had a history of recurrent depression with inpatient psychiatric admissions requiring ECT.

The patient’s family report him having always been different in personality. He struggled in social settings, found it hard to make friends, and had never been in a romantic relationship. He was obsessional about routine and was known as a child to pace and exhibit repetitive actions when anxious. He also had very specific and intense interests, such as obsessively learning the Latin names of plants. He had not previously been diagnosed with a childhood or developmental mental disorder. His only medical history consisted of a tonsillectomy and childhood scarlet fever. He took no regular medication, did not smoke cigarettes, and consumed no alcohol, and there was no recreational drug use. He was not known to the social care service and had no forensic history.

At the start of the current episode, the patient was referred for an urgent mental health consultation by his GP owing to concerns about his mental state over the preceding few weeks. He had retreated into his bedroom and was not washing himself or eating. He was noted to be unkempt, thin, and frail. He displayed objective signs of depression and reported feeling lonely. He stated that he had suicidal thoughts but denied intent. He was noted to speak quietly but was repetitive with short responses. He was not fully orientated to time or place, and his attention was notably poor when tested. He was admitted to a psychiatric ward for older people for a period of assessment. His GP found no acute physical cause for his symptoms, and on admission no abnormalities were detected on all initial investigations, including a battery of blood tests. There were no positive findings on physical examination and specifically no focal neurology. His observations showed hypertension with a blood pressure of 160/72 mmHg, but were otherwise unremarkable.

**Fig. 1:**
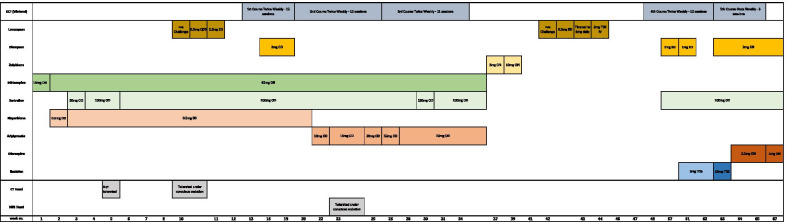
Timeline of treatment with electroconvulsive therapy (ECT) and medication during hospital admission, including timing of brain imaging

A long inpatient admission began, which would eventually last 16 months as summarized in a timeline shown in Fig. 1. Despite being admitted informally, he was soon deemed to lack mental capacity to consent to admission and was detained under the UK Mental Health Act 1983 (amended 2007). His clinical presentation included tearfulness, poor eye contact, reduced content of speech, frequent pacing, expression of thoughts of self-harm, and mimicking stabbing motions on himself. He wrote about a wish to die in his diary on the ward, although the content of this was disjointed. Collateral history from his family was received regarding his premorbid personality and level of functioning. It was concluded that he was experiencing severe depression on a background of autistic spectrum disorder. He was commenced on mirtazapine and then risperidone, which were titrated to effect, with sertraline added as a second antidepressant. During his initial assessment, the diagnosis of hebephrenic schizophrenia was considered. This was excluded after his family confirmed that prior to the current episode he had functioned relatively well, showering independently and preparing his own meals, for example, and his speech and behavior were not disorganized.

During the early weeks of his admission, the patient progressively became mute. He attempted to eat inanimate objects such as soap bars and continued to pace relentlessly. He also became intermittently incontinent of urine and feces. He was reviewed by the department for neurological sciences who excluded conditions such as motor neuron disease, and a negative autoantibody screen helped to exclude a diagnosis of limbic encephalitis. Dementia was suspected, and a brain computed tomography (CT) scan was attempted, but the patient did not tolerate this. Due to the importance of excluding an organic brain disorder, a repeat CT brain scan was organized but this time using conscious sedation with oral lorazepam (2 mg pre-scan). This resulted in an unexpected, sudden, and dramatic clinical response within minutes. The patient began speaking fluently and coherently for several hours, until his presentation of mutism, increased motor activity,and agitation returned. The use of lorazepam for conscious sedation, and the subsequent clinical response, was interpreted as a positive lorazepam challenge, and a syndrome of catatonia was confirmed. A trial of 0.5 mg lorazepam four times per day was commenced; however, the dose was reduced and then stopped altogether within days owing to severe sedation and frequent falls.

Following the unsuccessful trial of oral lorazepam, our patient was commenced on bilateral, twice weekly ECT (Somatics Thymatron System IV). After nine treatments, there were some signs of improvement, with brief and short episodes of speaking and increased writing in his diary. With ongoing ECT, his mutism became more intermittent, and this revealed disordered thought form. A diagnosis of schizophrenia was revisited as a possible contributing factor to his catatonia, with risperidone replaced by aripiprazole. Although still showing some signs of improvement after 24 ECT treatments, the patient continued to constantly pace and remained mute most of the time.

Notably, CT brain imaging and later magnetic resonance imaging (MRI) findings included mild generalized cerebral atrophy, including mild bilateral hippocampal volume loss, but no specific features of neurodegenerative disease, Creutzfeldt–Jakob disease, or cerebral amyloid angiopathy.

Following continued failure to significantly improve with ECT, antipsychotic medication was suspended owing to its possible effect on the poor response to ECT in catatonia, as has been reported previously [14, 15]. Sertraline and mirtazapine were also stopped to exclude serotonin syndrome as a potential cause of his restlessness. Zolpidem, which has previously been suggested as a treatment for catatonia [16], was then trialed for 2 weeks but provided no benefit. Following completion of 35 treatments of ECT, the patient remained mostly mute and displayed continued psychomotor agitation. However, his presentation then changed markedly to become rigid and unresponsive to external stimuli, demonstrating stupor. He continued to be mute and demonstrated repetitive teeth clenching (stereotypy) with negativism. He scored 16 on the Bush–Francis Catatonia Rating Scale. This deterioration in presentation prompted a further trial of lorazepam, initially given intravenously under close monitoring in a local general hospital.

As before, the first dose of lorazepam provided marked but temporary improvement in symptoms, as the patient started to talk fluently, and his rigidity disappeared. He was again trialed on regular intravenous then oral lorazepam, cautiously increased from 0.5 mg twice daily to 6 mg daily. Unfortunately, the patient developed aspiration pneumonia and remained in the general hospital for treatment of this. His prognosis was thought to be very poor, and his lorazepam was stopped owing to the risk of respiratory depression. He was deemed too unfit for anesthetic to undergo further ECT. At this point, he was transferred back to our psychiatric hospital for end-of-life care, with his family in agreement.

Somewhat remarkably, shortly after transfer, he became slightly more alert and was able to be fed by staff, although he remained largely stuporous. As a last resort, he was re-referred for further ECT twice weekly. He again showed initial noticeable improvement with a repeat Bush–Francis Catatonia Rating Scale scored at 8. We postulate that the improved effect of ECT at this time may have been related to previous cessation of concurrent antipsychotic medication. Nonmedical treatment was also offered through intensive physiotherapy, including hydrotherapy and passive stretching to reduce the risk of permanent strictures.

Despite a further 15 treatments of ECT, there was no sustained benefit in psychomotor presentation. It had already been established that our patient could not tolerate high-dose benzodiazepines. By this time, the patient was confined to his water chair and would communicate briefly verbally, but he still required full nursing care for toileting, feeding, changing, and transferring. He often looked anxious, and the team considered medication for symptomatic relief. Sertraline was restarted to treat potential underlying depression, and low-dose diazepam was used for anxiety, with olanzapine added as an adjunct. There was no change in presentation 1 month after cessation of ECT, and a best-interests decision was made with his family not to trial further treatment and to transfer him to a nursing home for full-time care.

The patient remained in a stable condition for many months in the nursing home, before sadly dying following sepsis due to bullous pemphigoid 2 years after his initial presentation.

## Discussion

Catatonia is a frequently reversible condition with prompt diagnosis and treatment. Our patient was sadly refractory to treatment with benzodiazepines and ECT, and we propose several important factors in this case that may have led to the poor outcome. These include his older age, delayed recognition of catatonia, comorbid autistic spectrum disorder, and sensitivity to benzodiazepine treatment. An obvious limitation to this case report is that we were unable to identify positive prognostic factors owing to the poor outcome for our patient.

Our patient’s initial presentation appeared to mimic depression, although an early clue for catatonia was increased motor activity preadmission. Numerous potential causes for catatonia were considered and investigated, including, but not limited to, severe depression, schizophrenia, dementia, limbic encephalitis, and other rarer neurological disorders. This diagnostic uncertainty led to a delay in recognition and treatment of catatonia. One small case series described delayed identification of catatonia in three elderly patients, all of whom had a poor outcome to treatment [11]. We also identified our patient’s reduced speech content as a factor that precluded prompt and thorough assessment of mental state. A retrospective study examining lorazepam treatment for catatonia showed that mutism and longer disease duration were predictors of poor outcome [17]. Furthermore, a long period of untreated catatonia, and chronic catatonic states, have been associated with a poorer response to benzodiazepines [6, 18].

Abnormal baseline social interaction and response to stress meant that our patient’s previously undiagnosed autistic spectrum disorder further complicated assessment of his psychomotor signs. The delayed recognition of catatonia in autistic spectrum disorder may in part be due to overlapping clinical features between both diagnoses, including posturing, stereotypies, and alterations in motor activity [19], as shown in our patient. In young adults with autism, it has been proposed that catatonia should be considered whenever there is a marked deterioration in movement, self-care, and pattern of activities [20]. Even if catatonia is recognized in autistic spectrum disorder, as it has been in up to 12% of individuals with autism between 17 and 40 years of age [20], this comorbidity is associated with a poorer response to treatment [19]. Indeed, a previous case series highlighted a very poor response to benzodiazepines in this patient group [21]. An important learning point from this case report is that catatonia should be considered when there is a deterioration in presentation for anyone with autistic spectrum disorder, although it should be remembered that treatment for catatonia in this group of patients is less likely to be successful.

We believe that the age of our patient contributed significantly to several factors relating to his outcome. Catatonia is not often recognized in older adults, despite its known prevalence [22, 23], and the way in which catatonia responds to treatment in older patients is poorly understood. One case series showed that older patients were less likely to respond to ECT than younger patients [15]. Older age also brings a much higher risk of adverse events when using benzodiazepines, including an increased risk of sedation, falls, and fractures [24–26]. There is also evidence that benzodiazepines relax the lower esophageal sphincter and potentially make aspiration more likely [27]. Our patient experienced severe adverse effects with lorazepam and developed aspiration pneumonia when higher doses were cautiously trialed. Overall, the use of benzodiazepines is more problematic in old age, meaning that outcomes may be poorer in older individuals with catatonia.

Our patient’s long admission, which included a range of interventions in different hospital settings, prompted reflection about several ethical and practical considerations. Given the poor outcome in our patient, and the poor prognosis of treatment of catatonia in old age overall, we recommend early discussion about appropriateness of treatment options. Specifically, a shorter treatment duration may be more ethical in this age group where circumstances may indicate a much poorer prognosis. Furthermore, there are practical considerations with regard to the location of treatment provision, with intravenous lorazepam usually only given in acute hospitals. Giving such high doses of sedating medication requires close monitoring for adverse effects, and logistical practices such as nurse-to-patient ratios and monitored beds should be considered.

## Conclusions

There remain significant limitations in our knowledge of how catatonia presents in old age. In this case report, the unsuccessful treatment of catatonia in an elderly patient highlights several clinical implications for this age group. We recommend early consideration of catatonia in old-age mental health services and acute medical services if relevant clinical features are present. Once catatonia has been recognized, it is important to ensure the early use of ECT owing to reduced tolerability of benzodiazepines in the elderly, as we know that prompt treatment improves outcomes in this age group. We recommend that further exploration of the presentation, course, and treatment of catatonia in the elderly is essential to improve clinical outcomes in this population.

## Data Availability

Not applicable.

## Abbreviations

CT: Computed tomography
ECT: Electroconvulsive therapy
GABA: Gamma-aminobutyric acid
GP: General practitioner
MRI: Magnetic resonance imaging

## References

[1] Kraines SH. Bell’s mania. Am J Psychiatry. 1934;91(1):29–40.

[2] Carroll BT. Kahlbaum’s catatonia revisited. Psychiatry Clin Neurosci. 2001;55(5):431–6. 10.1046/j.1440-1819.2001.00887.x 11555337

[3] Tandon R, Heckers S, Bustillo J, Barch DM, Gaebel W, Gur RE, *et al*. Catatonia in DSM-5. Schizophr Res. 2013;150(1):26–30. 10.1016/j.schres.2013.04.034 23806583

[4] Rasmussen SA, Mazurek MF, Rosebush PI. Catatonia: our current understanding of its diagnosis, treatment and pathophysiology. World J Psychiatry. 2016;6(4):391–8. 10.5498/wjp.v6.i4.391 PMC5183991 28078203

[5] Kirkhart R, Ahuja N, Lee JW, Ramirez J, Talbert R, Faiz K, *et al*. The detection and measurement of catatonia. Psychiatry (Edgmont (Pa Township)). 2007;4(9):52–6. PMC2880943 20532121

[6] Sienaert P, Dhossche DM, Vancampfort D, De Hert M, Gazdag G. A clinical review of the treatment of catatonia. Front Psychiatry. 2014;5:181. 10.3389/fpsyt.2014.00181 PMC4260674 25538636

[7] American Psychiatric Association. Diagnostic and statistical manual of mental disorders. 5th ed. 2013. 10.1176/appi.books.9780890425596

[8] Pelzer AC, van der Heijden FM, den Boer E. Systematic review of catatonia treatment. Neuropsychiatr Dis Treat. 2018;14:317–26. 10.2147/NDT.S147897 PMC5775747 29398916

[9] Bush G, Fink M, Petrides G, Dowling F, Francis A, Catatonia I. Rating scale and standardized examination. Acta Psychiatr Scand. 1996;93(2):129–36. 10.1111/j.1600-0447.1996.tb09814.x 8686483

[10] Ellul P, Choucha W. Neurobiological approach of catatonia and treatment perspectives. Front Psychiatry. 2015;6:182. 10.3389/fpsyt.2015.00182 PMC4689858 26733892

[11] Swartz C, Galang RL. Adverse outcome with delay in identification of catatonia in elderly patients. Am J Geriatr Psychiatry. 2001;9(1):78–80. 11156756

[12] Takata T, Takaoka K, Fujigaki M. Catatonia in the elderly. Int J Psychiatry Clin Pract. 2005;9(4):230–7. 10.1080/13651500500240670 24930919

[13] Alisky JM. Lorazepam-reversible catatonia in the elderly can mimic dementia, coma and stroke. Age Ageing. 2007;36(2):229. 10.1093/ageing/afl168 17255087

[14] Hawkins JM, Archer KJ, Strakowski SM, Keck PE. Somatic treatment of catatonia. Int J Psychiatry Med. 1995;25(4):345–69. 10.2190/X0FF-VU7G-QQP7-L5V7 8822386

[15] van Waarde JA, Tuerlings JH, Verwey B, van der Mast RC. Electroconvulsive therapy for catatonia: treatment characteristics and outcomes in 27 patients. J ECT. 2010;26(4):248–52. 10.1097/YCT.0b013e3181c18a13 19935090

[16] Peglow S, Prem V, McDaniel W. Treatment of catatonia with zolpidem. J Neuropsychiatry Clin Neurosci. 2013;25(3):E13. 10.1176/appi.neuropsych.11120367 24026726

[17] Tibrewal P, Narayanaswamy J, Zutshi A, Srinivasaraju R, Math SB. Response rate of lorazepam in catatonia: a developing country’s perspective. Progress Neuro Psychopharmacol Biolog Psychiatry. 2010;34(8):1520–2. 10.1016/j.pnpbp.2010.08.017 20804808

[18] Ungvari GS, Chiu HF, Chow LY, Lau BS, Tang WK. Lorazepam for chronic catatonia: a randomized, double-blind, placebo-controlled cross-over study. Psychopharmacology. 1999;142(4):393–8. 10.1007/s002130050904 10229064

[19] Ghaziuddin M, Quinlan P, Ghaziuddin N. Catatonia in autism: a distinct subtype? J Intellect Disabil Res. 2005;49(Pt 1):102–5. 10.1111/j.1365-2788.2005.00666.x 15634317

[20] Kakooza-Mwesige A, Wachtel LE, Dhossche DM. Catatonia in autism: implications across the life span. Eur Child Adolesc Psychiatry. 2008;17(6):327–35. 10.1007/s00787-008-0676-x 18427869

[21] Wachtel LE. Treatment of catatonia in autism spectrum disorders. Acta Psychiatr Scand. 2019;139(1):46–55. 10.1111/acps.12980 30506668

[22] Jaimes-Albornoz W, Serra-Mestres J. Prevalence and clinical correlations of catatonia in older adults referred to a liaison psychiatry service in a general hospital. Gen Hosp Psychiatry. 2013;35(5):512–6. 10.1016/j.genhosppsych.2013.04.009 23684045

[23] Kaelle J, Abujam A, Ediriweera H, Macfarlane MD. Prevalence and symptomatology of catatonia in elderly patients referred to a consultation-liaison psychiatry service. Australas Psychiatry. 2016;24(2):164–7. 10.1177/1039856215604998 26400451

[24] Hill KD, Wee R. Psychotropic drug-induced falls in older people: a review of interventions aimed at reducing the problem. Drugs Aging. 2012;29(1):15–30. 10.2165/11598420-000000000-00000 22191720

[25] Bakken MS, Engeland A, Engesaeter LB, Ranhoff AH, Hunskaar S, Ruths S. Risk of hip fracture among older people using anxiolytic and hypnotic drugs: a nationwide prospective cohort study. Eur J Clin Pharmacol. 2014;70(7):873–80. 10.1007/s00228-014-1684-z PMC4053597 24810612

[26] Xing D, Ma XL, Ma JX, Wang J, Yang Y, Chen Y. Association between use of benzodiazepines and risk of fractures: a meta-analysis. Osteoporos Int. 2014;25(1):105–20. 10.1007/s00198-013-2446-y 24013517

[27] Rushnak MJ, Leevy CM. Effect of diazepam on the lower esophageal sphincter. A double-blind controlled study. Am J Gastroenterol. 1980;73(2):127–30. 7395839

